# TMT labeled comparative proteomic analysis reveals spleen active immune responses during *Clostridium perfringens* type C infected piglet diarrhea

**DOI:** 10.7717/peerj.13006

**Published:** 2022-04-04

**Authors:** Xiaoli Wang, Xiaoyu Huang, Qiaoli Yang, Zunqiang Yan, Pengfei Wang, Xiaoli Gao, Ruirui Luo, Shuangbao Gun

**Affiliations:** 1 College of Animal Science and Technology, Gansu Agricultural University, Lanzhou, Gansu, China; 2 Guizhou Institute of Prataculture, Guizhou Academy of Agriculture Science, Guiyang, China; 3 Gansu Research Center for Swine Production Engineering and Technology, Lanzhou, Gansu, China

**Keywords:** TMT labeling, Proteome, Clostridium perfringens type C, Piglet diarrhea, Immune Response, Spleen

## Abstract

**Background:**

*Clostridium perfringens* (*C. perfringens*) type C is the principal pathogenic clostridia of swine, frequently causing hemorrhagic diarrhea, even necrotic enteritis in piglets, leading to severe economic loss for swine industr ies worldwide. However, there are no specific and effective prevention measures. Therefore, clarifying the molecular mechanisms of hosts against pathogenesis infection is very important to reduce the incidence of *C. perfringens* type C infected piglet diarrhea disease.

**Methods:**

We performed an TMT labeling-based quantitative spleen proteomic analysis of the control group (SC), tolerance group (SR) and susceptible group (SS) to identify the differentially expressed proteins (DEPs), and screened potential molecular markers of piglet spleen tissues in response to *C. perfringens* type C infection.

**Results:**

In this study, a total of 115, 176 and 83 DEPs were identified in SR vs SC, SS vs SC, and SR vs SC, respectively, which may play the important regulatory roles in the process of piglet spleens in response to*C. perfringens* type C-infected diarrhea diseases. GO enrichment analysis revealed that the DEPs were mostly significantly enriched in acute inflammatory response, defense response, antimicrobial response, transporter activity, cellular metabolic process and so on, and KEGG pathway enrichment analysis showed that the significantly enriched immune related pathways of the PPAR signaling pathway, IL-17 signaling pathway, antigen processing and presentation, which hints at the immune defense process of piglet spleen against *C. perfringens* infection. This study helps to elucidate the protein expressional pattern of piglet spleen against *C. perfringens* type C-infected diarrhea disease, which can contribute to the prevention and control for pig diarrhea disease and the further development of diarrhea resistant pig breeding.

## Introduction

*Clostridium perfringens* (*C. perfringens*) is the most widespread of pathogenic bacteria in environment (Labbe & Vijay, 2017), which commonly caused several diseases, such as diarrhea, intestinal inflammation and necrotic enteritis in domestic animals and humans (Shrestha, Uzal & McClane, 2018; Uzal et al., 2014). Statistically, *C. perfringens* is the important reason of food poisoning, and causes nearly 1 million cases of human foodborne illness with symptoms of abdominal cramps and diarrhea yearly in the United States (Grass, Gould & Mahon, 2013; Miyamoto & Nagahama, 2016).

Usually, *C. perfringens* has been divided into seven subtypes, designated A, B, C, D, E, F and G (Rood et al., 2018). *C. perfringens* type C is a dominant factor of piglet diarrhea, mainly for piglets under 7-day age, with extremely high morbidity (60∼80%) and mortality (80∼100%), resulting in great economic loss for pig industries (Park et al., 2016). *C. perfringens* type C can produce variety lethal toxins in the intestines of piglets, and these toxins are transferred to the spleen and other organs causing systemic damage through blood and lymph circulation (Uzal et al., 2015). Studies have reported that *C. perfringens* type C infection causes systemic immune and inflammatory disease, with the obvious clinical symptoms of acute hemorrhagic diarrhea, slow growth and death in piglets (Huang et al., 2019). *C. perfringens* type C is increasingly considered to the pathogenic bacteria of pig source for high morbidity in neonatal piglet diarrhea (Songer, 2010; Chan et al., 2012).

Currently, although some progress has been made in molecular epidemiology, diagnosis, prevention and treatment for *C. perfringens* infection (Smits et al., 2016), much more is urgent to illustrate the comprehensive gene-encoding protein function at the omics level, to explore the mechanism of immune regulation and potential activated signaling pathway, and to find better ways of prognosis and treatment for *C. perfringens* infection.

Comparative proteomics is a powerful approach to screen proteomic difference comprehensively, which can identify and quantify thousands of proteins simultaneously (Chen et al., 2019). By differentiating the differential protein expression between two experiment groups or more, it can discover the function of proteins, the potential interactions between proteins and the mining of new proteins from protein expression changes, which is critical to explore and understand protein kinetics and mechanism of biological function at the molecular level (Ruiz et al., 2016a; Ruiz et al., 2016b; Su et al., 2017). Li et al. (2016) had studied the pathogenesis mechanism of pigs infected by porcine epidemic diarrhea virus (PEDV) YN13/YN144 by iTRAQ labeling and LC-MS/MS, and identified the differently expressed proteins (DEPs) proposed heterogeneous nuclear ribonucleoprotein A1 (hnRNPA1), eukaryotic initiation factor 4G1 (eIF4G1), and heat shock protein (HSP) family could be responsible for the pathogenicity differences in piglets after infection (Li, et al. 2016).

Zhang et al. (2013) had studied the protein expressions of porcine transmissible gastroenteritis virus (TGEV) infected pig testicle ST cells by wo-dimensional difference gel electrophoresis, and found that 33 DEPs (23 upregulation and 10 downregulation) were involved in the regulation processes by cellular structure and integrity, RNA processing, protein biosynthesis and modification, vesicle transport, signal transduction, and the mitochondrial pathway (Zhang et al. 2013). Ma et al. (2014) used iTRAQ and LC-MS/MS techniques to study the proteomic of ST cells with infected and uninfected TGEV, and a total of 146 DEPs and 219 DEPs were identified in TGEV infected ST cells 48 h and 64 h, respectively, suggesting the longer infection time, the more significant changes of protein expressions in ST cells (Ma et al., 2014).

However, there is no study to evaluate the regulatory mechanism of protein level in piglet diarrhea caused by *C. perfringens* type C infection.

Spleen is the important immune organ for the vital role in capturing and destroying pathogens, and inducing body adaptive immune responses in host immune response system (Mebius & Kraal, 2005; Truong, Hong & Lillehoj, 2015). Moreover, spleen includes the rich immune cells, such as macrophages, dendritic cells (DCs) and monocytes, which can not only recognize pathogens and cell stress, remove dead cells and foreign materials, but also regulate tissue homeostasis and inflammatory responses to form adaptive immunity (Lewis, 2019). Research has also provided the certain protection against to *C. perfringens* infection by modulating innate and adaptive immunity (Hong et al., 2012).

Our previous work had been reported that *C. perfringens* type C infection caused to the significantly differential expression of immune genes (*TLR4*, *TNF- α* and *NF- κB*), cytokines and proinflammatory factors (IFN- *γ*, IL-6 and IL-12) and serum immunoglobulin (Shi et al., 2019), and the abnormal obviously histological damage of spleens and small intestines of piglets, such as inflammatory cell and neutrophile granulocytes infiltration (Yan et al., 2018; Yan et al., 2019), these all demonstrated that *C. perfringens* type C infection caused to the significantly immune and inflammatory responses of piglets during bacterial infected diarrhea. Therefore, in this study, we adopted TMT-labeled LC-MS/MS to profile global spleen proteome of tolerance and susceptibility piglet spleen tissues of *C. perfringens* type C infection, screening differentially expressed proteins, discussing functional protein of mediating *C. perfringens* type C -infected piglet diarrhea, which may contribute to further study on the prevention and control for pig diarrhea disease and the further development of diarrhea resistant pig breeding.

## Materials & Methods

### Ethics statement

This study was carried out in accordance with the recommendations of Institutional Animal Care and Use Committee (IACUC) of Gansu Research Center for Swine Production Engineering and Technology. The protocol was approved by the College of Animal Science and Technology, Gansu Agricultural University.

### Animal infection experiment and sample collection

The animal experiment progress was consistent as the description of Huang et al. (2019), the detail was as following: thirty healthy 7-day-old piglets (Landrace × Yorkshire, Xitai breeding co., LTD. Gansu Province) were chosen with detected serum antibodies negatively for *E. coli* (enterotoxin and K88), *Salmonella* (typhimurium, typhisuis and choleraesuis) and *C. perfringens* type C *β* toxin antibodies by enzyme-linked immunosorbent assay (ELISA).

The *C. perfringens* type C strain (CVCC 2032, China Veterinary Culture Collection Center) was shaking cultured 16 h at 37 °C in the bouillon culture-medium (HopeBio, Qingdao, China) before used for infection. The colony-forming units (CFUs) of *C. perfringens* type C was determined by plate colony counting method, and finally an expected concentration of 1 × 10^9^ CFU/mL *C. perfringens* type C medium was used to inoculate piglets.

Twenty-five piglets were randomly selected to suffer oral inoculate administration of 1 × 10^9^ CFU/mL *C. perfringens* type C medium, the remaining 5 piglets were orally inoculated sterile medium as control group (SC), the experiment lasted for 5 days for the research report (Songer & Uzal, 2005) and the result of our pre-experiment. During the 5-day experimental period, all piglets were housed separately by special environmental control equipment, bodyweight, mental state, shape and color of feces and degree of diarrhea for each piglet were detailly observed and recorded detailly in every day. The degree of diarrhea was evaluated according to the diarrhea scoring standard of piglets (Huang et al., 2019): 0 point for strip or granular feces; one point for soft forming; two points for thick and unformed feces; three points for liquid and watery excrement.

At the end of the test, the total diarrhea scores of each piglet were calculated by adding each defecation score of each piglet during the test period. Finally, 3 piglets with the highest and lowest total diarrhea scores were considered as the susceptible group (SS) and t the tolerance group (SR) (Fig. S1). In general, SS represents in sensitive to *C. perfringens* infection with serious diarrhea, and SR represents which resistant to *C. perfringens* infection with mild diarrhea.

Finally, nine piglet spleen tissues of SS, SR and SC groups were rapidly collected without RNA enzyme and washed with PBS buffer frozen by liquid nitrogen and transferred to −80 °C for storage.

### Protein extraction

Spleen samples were added and mixed by lysis buffer (8 M urea, 1% Protease Inhibitor Cocktail) for standing still for 30 min, then followed by sonication on ice three times using a high-intensity ultrasonic processor (Scientz). After fully lysing on ice 30 min, the remaining precipitated debris was removed by centrifugation at 12,000 g at 4 °C for 10 min. The supernatant was collected and protein concentration was determined by BCA kit (Beyotime biotechnology, Shanghai, China).

### Trypsin digestion

Protein solution was reduced with 5 mM dithiothreitol for 30 min at 56 °C and then alkylated with 11 mM iodoacetamide for 15 min. Then, the protein sample was diluted by adding 100 mM TEAB to urea concentration less than 2 M for trypsin digestion with first digestion of 1:50 trypsin-to-protein ratio overnight and the 4 h second digestion of 1:100 ratios. Approximately 100 µg protein for each sample was used for the following experiments.

### TMT labeling

After trypsin digestion, the peptide was desalted by Strata X C18 SPE column (Phenomenex) and vacuum-dried. Briefly, peptide was reconstituted in 0.5M TEAB and processed according to the manufacturer’s protocol for TMT kit (Thermo Fisher Scientific, Waltham, Massachusetts). Then, one unit of TMT reagent (defined as the amount of reagent required to label 100 µg of protein) was thawed and reconstituted in 24 µL ACN. Finally, peptide mixtures were incubated for 2 h at 37 °C, following desalting and drying by vacuum centrifugation.

### Peptide fractionation by high-performance liquid chromatography (HPLC)

The tryptic peptides were fractionated by high pH reverse-phase HPLC using C18 column (5 µm particles, 10 mm ID, 250 mm length). According the standard analytical procedure, peptides were firstly separated with a gradient of 8% to 32% acetonitrile (pH 9.0) over 60min into 60 fractions, then combined into 18 fractions and 6 fractions, respectively, and dried by vacuum centrifugation.

### LC-MS/MS analysis

The tryptic peptides were dissolved in 0.1% formic acid (solvent A), directly loaded onto a home-made reversed-phase analytical column (15 cm length, 75 µm ID). The gradient was increased from 6% to 23% solvent B (0.1% formic acid in 98% acetonitrile) over 26min, from 23% to 35% in 8min and climbing to 80% in 3min then holding at 80% for the last 3min. The constant flow rate was 400 nL/min with the EASY-nLC 1000 UPLC system.

Intact peptides were detected in the Orbitrap at a resolution of 70,000. Peptides were selected for MS/MS using a normalized collision energy setting of 28; the Peptides and peptide fragments were detected in the Orbitrap at a resolution of 17,500. A data-dependent procedure that MS scan were followed by 20 MS/MS scans was applied for the top 20 precursor ions above a threshold ion count of 1.5E4 in the MS survey scan with 15.0s dynamic exclusion. An electrospray voltage of 2.0 kV was applied. Automatic gain control (AGC) was used to prevent overfilling of the iontrap; 5E4 ions were accumulated for generation of the MS/MS spectra. For MS scans, the m/z scan range was 350 to 1,600. Data are available *via* ProteomeXchange with identifier PXD030745.

### Protein identification and analysis

Maxquant software (v 1.6.4) was used for MS/MS data peptide identification and quantitation against UniProt Proteomes-*Sus scrofa* (UP000008227) with the following parameters: enzyme—Trypsin, peptide mass tolerance—± 10 ppm, fragment mass tolerance—± 0.02 Da, max missed cleavage—2, fixed modification—carbamidomethyl (C), variable modification—oxidation (M) and deamidated (NQ), quantification method—TMT10 plex report ion MS2, database pattern—decoy. Maximum false discovery rates (FDRs) were set to 1% for peptide and protein identification.

### Screening differentially expressed proteins

The MS data validation was by mass error distribution of all identified peptides and peptide length distribution. Firstly, we checked the mass error of all the identified peptides. The distribution of mass error is near zero and most of them are less than 0.02Da which means the mass accuracy of the MS data fit the requirement. Secondly, the lengths of most peptides were distributed between 8 and 20, which agree with the property of tryptic peptides, meaning sample preparation reach the standard. The proteins with a log 2 (fold change) ≥1.3 and a *P* value < 0.05 were considered as differentially expressed protein.

### Quantification validation of MS data

Only peptides unique for a certain protein were considered for TMT relative quantification, which was normalized using the average ratio of all the unique peptides in each sample. A two-tailed Fisher’s exact test was employed to test the enrichment of the differentially expressed protein against all identified proteins. A corrected *P* value < 0.05 was considered significant. For further hierarchical clustering based on different protein functional classification, the cluster membership was visualized by a heat map using the “heatmap.2” function from the “gplots” R-package.

### Functional enrichment analysis

The different expressed proteins were performed to Gene Ontology (GO) enrichment analysis and Kyoto Encyclopedia of Genes and Genomes (KEGG) pathways analysis using DAVID (version 6.7). The protein functional annotation was derived from the UniProt-GOA database (http://www.ebi.ac.uk/GOA/) (Ashburner et al., 2000). The functional annotation and descriptions of identified protein domains were annotated by InterProScan (a sequence analysis application) based on protein sequence the InterPro (http://www.ebi.ac.uk/interpro/) domain database. The identified proteins domain functional description was annotated by InterProScanbased (http://www.ebi.ac.uk/interpro/) on protein sequence alignment.

### Protein-protein interaction network

All differentially expressed proteins interactions, were analyzed against the *Sus scrofa* database by STRING version 10. Only the confidence score ≥ 0.7 (high confidence) of interactions was fetched. Interaction network form STRING was visualized in R package “networkD3”. Cytoscape was used for the visualization of the networks.

### Statistical analysis

One-way ANOVA and Duncan’s multiple comparisons in SPSS 26.0 software were used to test the significance of difference between diarrhea scores of piglets, the weight of piglets before euthanasia, and the weight of heart, liver, spleen, lung and kidney after euthanasia in the groups of SR, SS and SC, Differences with a *P* < 0.05, the log2-transformed ratio is larger than 1.3 were considered as significant difference. Results were shown as the means and standard errors (mean ± SE).

## Results

### Phenotypic characterization of piglets infected with *C. perfringens* type C

**Figure 1:**
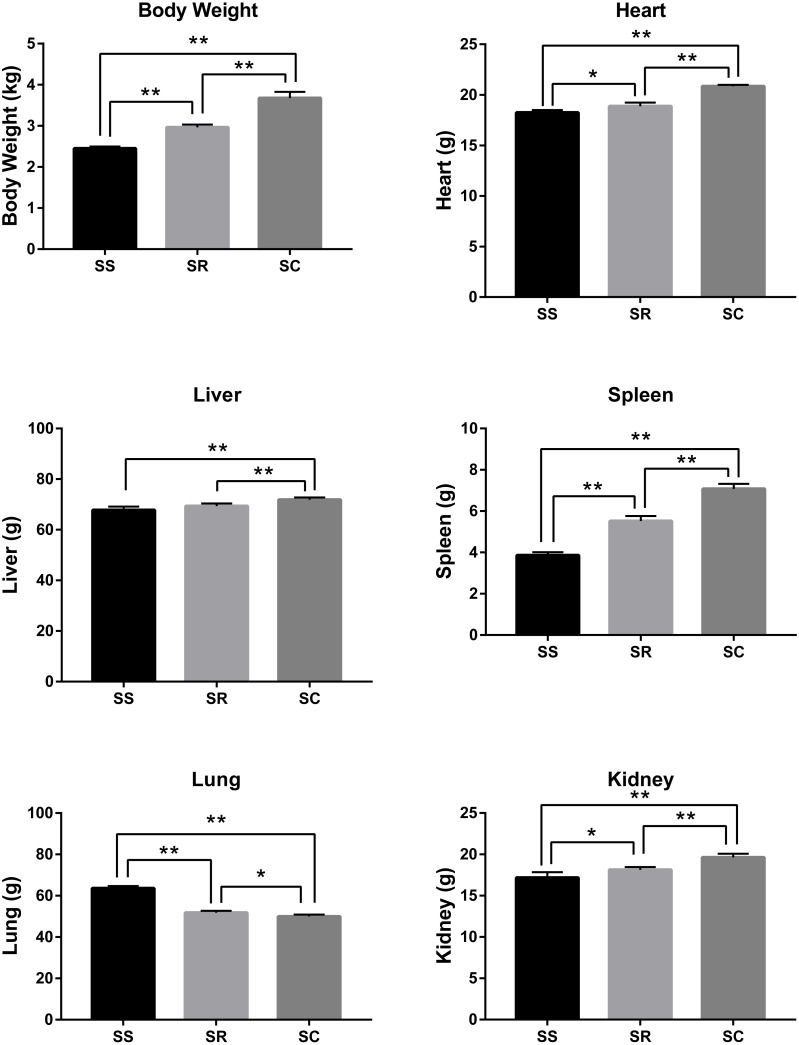
The difference of bodyweight, heart, liver, spleen, lung and kidney of the piglets in SS, SR and SC groups. * *P* < 0. 05, representing meaningful difference; ** *P* < 0.01, representing a significant difference.

After the infection of *C. perfringens* type C, the inoculated piglets presented the varying degrees of diarrhea symptom. As shown in Fig. 1, the bodyweight, heart, liver, spleen, and kidney of inoculated piglets in the SS and SR groups were significantly lower than those of control group (SC) (*P* < 0.01). Meanwhile, the weights of body and heart, spleen, kidney and liver in SS group were the lightest. In addition, lung weight in SS and SR groups were significantly higher than that in SC group (*P* < 0.01, *P* < 0.05), and SS group has the heaviest lung weight.

### Quantitative identification of spleen proteins for piglets

To comprehensively profile the landscape of spleen protein expression before and after *C. perfringens* infection, we had identified and quantified the differential proteome dynamics in three replicates during the *C. perfringens* infection. According to the experimental workflow (Fig. S1), we achieved comprehensive proteome profiling with deep coverage using TMT-labeled LC-MS/MS combined with pre-fractionation by HPLC, allowing for identification and quantification simultaneously. In total, 6,145 proteins can be identified, among which 4,958 proteins were quantified in the global proteome (Supplemental Table S1). In addition, many uncharacterized proteins were identified according to bioinformatics comparison results and the comprehensive annotation (Supplemental Table S2).

### QC validation of MS data

To validate the quality of MS and MS/MS profiling, the mass error of all the identified peptides were checked, results demonstrated that the mass accuracy of the MS data and sample preparation fit the experiment requirement standard. The repeatability among the three groups was assessed by principal component analysis (PCA), relative standard deviation (RSD) and Pearson correlation coefficient, results indicated that there were the higher aggregation degree and correlation among SS, SR and SC groups (Figs. S2A, S2B, S2C), which proved that the proteomic analysis was robust and all data would be used for the following analysis with high quality.

### Differentially quantified proteins identified among SC, SR and SS

**Figure 2:**
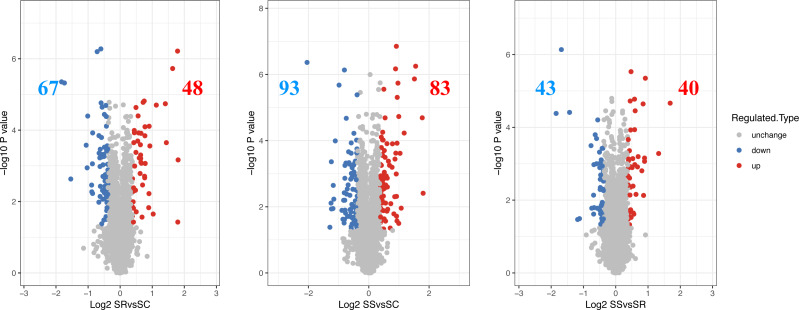
The differential expressed proteins identified in piglet spleen of SR *vs* SC, SS *vs* SC and SS *vs* SR. Red represents the up-regulated proteins, blue represents the down-regulated proteins, grey represents the nondifferential proteins.

The differentially expressed proteins and specific number for differential proteins between SR, SS and SC were analyzed. The proteins exhibiting a log 2 (fold change) > 1.3 and a *P* value < 0.05 were regarded as DEPs, then a total of 115 DEPs were identified in SR *vs* SC, of which 48 were up-regulated proteins and 67 were down-regulated proteins, 176 DEPs were identified with 83 up-regulated proteins and 93 down-regulated proteins in SS *vs* SC, 83 DEPs were identified, with 40 were up-regulated proteins and 43 down-regulated proteins in SR *vs* SC (Fig. 2, Table S3). The above differentiation of protein expression indicated molecular changes and potential functional transformation underlined *C. perfringens* infected piglets.

### Bioinformatic analysis of differentially expressed proteins

**Figure 3:**
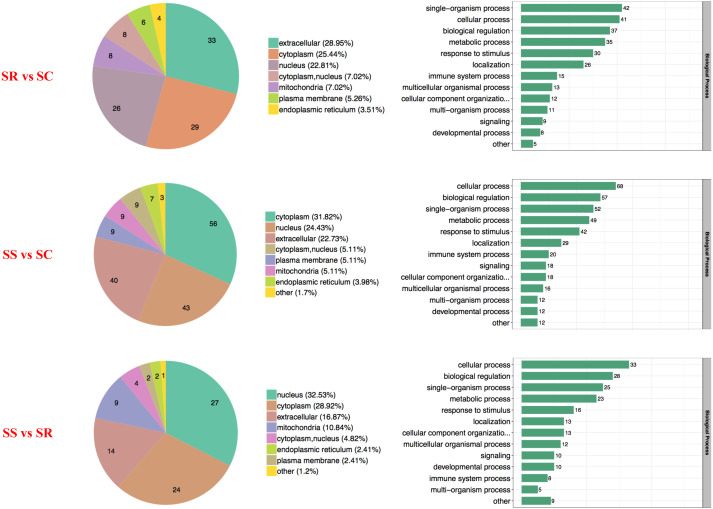
Wolfpsort-based subcellular localization prediction of the differentially abundant proteins of SR, SS and SC groups.

The proteins with diverse biological processes and distinct subcellular locations performed significantly different molecular functions. There was a wide range of functional distribution of the DEPs among SS, SR and SS groups for each module, the three top were mainly located in extracellular, cytoplasm and nucleus, and detailly, extracellular enriched the most differential proteins for SR *vs* SC, cytoplasm for SS *vs* SC, and nucleus for SS *vs* SR (Fig. 3).

**Figure 4:**
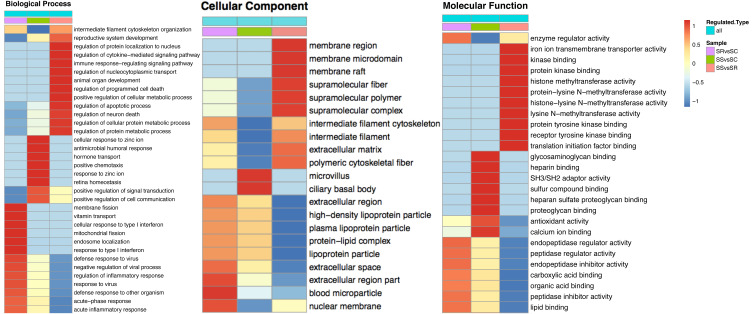
Classification of the DEPs of SR, SS and SC groups. Biological processes (A), cellular components (B) and molecular functions (C) of GO annotation based on function classification.

The heatmap comparisons of SS *vs* SC, SR *vs* SC, SS *vs* SR groups were shown in Fig. 4 and Table S4, which divided into biological process, cellular component and molecular function. The DEPs with same expression pattern were clustered together.

### Functional enrichment analysis of DEPs

**Figure 5:**
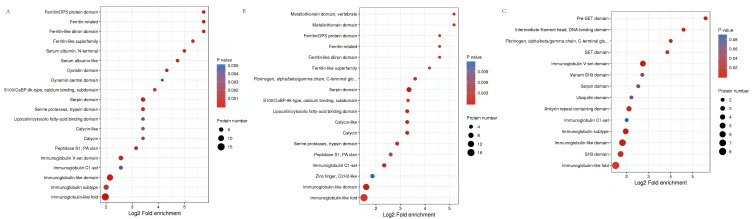
The bubble pattern of GO functional enrichment analysis of the DEPs identified in piglet spleen among (A) SR *vs* SC, (B) SS *vs* SC and (C) SS *vs* SR comparison. The heat map represented the enrichment results of different groups horizontally, and the longitudinal description of GO analysis, including biological process, cellular component and molecular function. The color blocks corresponding to the differentially expressed proteins and functional descriptions in different groups indicate the degree of enrichment. Red indicates strong enrichment and blue indicates weak enrichment.

To explore the function of DEPs among SS, SR and SC groups, GO and KEGG functional enrichment analysis were performed. GO catalogs analysis of the DEPs among SR *vs* SC, SS *vs* SC and SR *vs* SS groups revealed that the most significantly enriched biological processes as acute inflammatory response, defense response to bacterium, leukocyte aggregation, defense response, cellular oxidant detoxification, antimicrobial response, positive regulation of cellular metabolic process, the main cellular component functions as extracellular space, protein-lipid complex, and the main molecular functions as tyrosine kinase binding, kinase binding, iron ion transmembrane transporter activity, Toll-like receptor 4 binding, RAGE receptor binding, nucleosomal DNA binding (Fig. 5, Table S4).

Specially, in the SS *vs* SR groups, the biological process included many immune and metabolism associated functions, such as defense response to virus, inflammatory response, regulation of response to virus immune response, defense response to other organism, antimicrobial humoral response, regulation of cytokine-mediated signaling pathway, regulation of apoptotic process and so on. The cellular components were mainly cell and organelle. And the molecular function mainly enriched in binding and enzyme catalytic activity of methyltransferase, enzyme regulator, peptidase regulator and endopeptidase inhibitor, translation initiation factor receptor binding.

**Figure 6:**
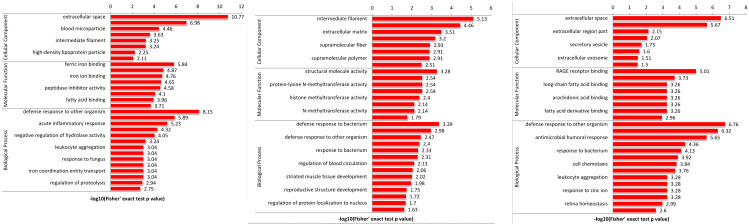
Protein domain analysis of the DEPs identified in piglet spleen among SR *vs* SC (A), SS *vs* SC (B) and SS *vs* SR (C) comparison groups.

Functional domains related to immunoglobulin-like fold, MHC classes I/II-like antigen recognition protein, calcium S100/CaBP-9k-type, Ferritin/DPS protein domain, Fibrinogen were conspicuously enriched in these DEPs (Fig. 6, Table S5).

**Figure 7:**
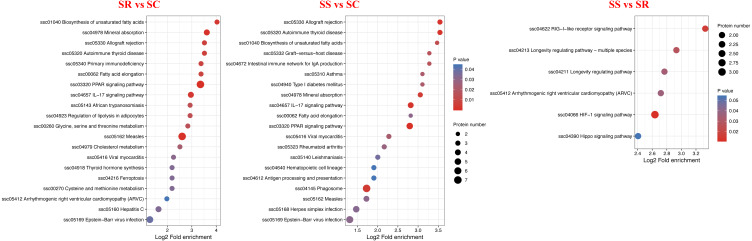
KEGG pathway enrichment analysis of the DEPs of (A) SR *vs* SC, (B) SS *vs* SC and (C) SS *vs* SR comparison groups. Red indicates strong enrichment and blue indicates weak enrichment.

KEGG analysis was performed to investigate the enriched pathways participated by the DEPs. A total of 30 KEGG signaling pathways were enriched among them, detailly, 13 signaling pathways for SR *vs* SC, 20 signaling pathways for SS *vs* SC and 5 signaling pathways for SS *vs* SR (Fig. 7, Table S6). In which, PPAR signaling pathway, IL-17 signaling pathway were highly enriched in SR *vs* SC and SS *vs* SC after *C. perfringens* infection, antigen processing and presentation, intestinal immune network for IgA production, hematopoietic cell lineage was specifically significantly in SS *vs* SC, RIG-I-like receptor signaling pathway were specifically significantly enriched in SS *vs* SR, these identified significantly signaling pathways were involved in the process of piglet spleen immune responses against *C. perfringens* infection.

### Analysis of protein interaction network

**Figure 8:**
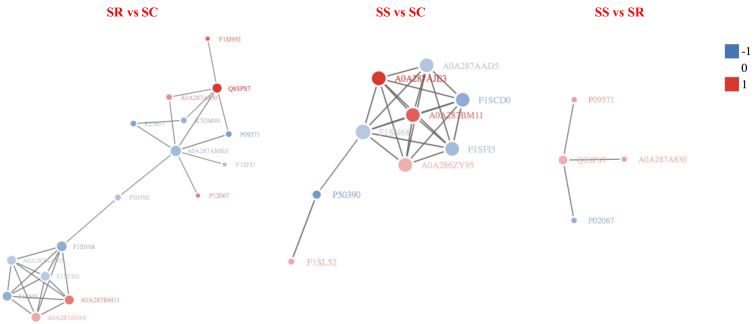
The protein-protein interaction network of DEPs among SR, SS and SC groups infected by *C. perfringens* type C. The network nodes represent numbers of proteins (up regulated in red; down regulated in green). Rounded rectangles represent candidate biological processes.

In order to clearly display the interactions between proteins, a network constitute of top 50 closest interactions proteins of functional protein-protein interactions was built using STRING v.10.0 online software against the *Sus scrofa* database (Ashburner et al., 2000). A total of 34, 47 and 10 known or predicted interactions (PPI enrichment *P*-value < 1.0e^−16^) were formed among DEPs in the PPI network (Fig. 8). The prediction of the protein interaction network of DEPs showed that P09571, A0A287AMK0, A0A287AQG3, C3S7K6, Q6S4N2, F1RQW9, and Q8SPS7 all had the pivotal role in each PPI network. The highest number of interactions was observed for Fibronectin 1 (FN1), Serum albumin (Manyes et al., 2014).

## Discussion

Piglet Diarrhea is an important factor affecting the healthy development of the pig industries worldwide (Zhao et al., 2016), *C. perfringens* type C has become the increasing threaten bacterial infections to lead pig diarrhea with characteristic of high morbidity and mortality, especially in newborn and suckling piglets (Uzal & McClane, 2011), even in domestic animals and humans (Diab et al., 2016), resulting in great economic loss. Our previous studies found that *C. perfringens* type C infection caused to the significantly up-regulations of immune genes *TLR4*, *TNF-α* and *NF-κB*, inflammatory cytokine factors IFN- *γ*, IL-1*β*, IL-6 and IL-12, and serum immunoglobulin IgA and IgG in tolerance and susceptible piglets (Shi et al., 2019). Histopathological findings the morphology of the intestines and spleens of piglets infected by *C. perfringens* type C were abnormal obviously, such as infection caused edema of the lamina propria and submucosa, inflammatory cell and neutrophile granulocytes infiltration in piglet intestines (Yan et al., 2019), and infiltration with neutrophilic granulocytes in the red pulp of spleen tissues (Yan et al., 2018). These studies had provided that there were significantly immune and inflammatory responses of piglet spleen in response to *C. perfringens* type C infection. However, it is still limited that the molecular mechanism of piglet spleen immune responses against to *C. perfringens* type C-infected diarrhea disease, which can improve to understand mechanism of piglet resistance to *C. perfringens* type C infection and may help to prevent and control pig bacterium diarrhea. Therefore, we had comprehensively profiled and characteristed the spleen proteomic dynamics of piglet against to *C. perfringens* infection by the TMT-labeled LC-MS/MS combined with pre-fractionation by HPLC.

In this study, a total of 6,145 identified proteins, including 4,958 quantified proteins were in piglet spleen tissues from SR, SS and SC groups suffered from *C. perfringens* infection. Differential expression analysis totally identified 115 DEPs (48 upregulated and 67 down-regulated proteins) identified in SR *vs* SC group, 176 DEPs (83 upregulated and 93 down-regulated proteins) in SS *vs* SC group, 83 DEPs (40 upregulated and 43 downregulated proteins) in SR *vs* SC group, in which, some immune-related proteins also identified, including P80310 (coded by *S100A12*) and C3S7K6 (coded by *S100A9*) both up-expressed in SR *vs* SC and SS *vs* SC groups, Q764N2 (coded by *CD3D*) up-expressed in SR *vs* SC group, F1SNY4 (coded by *TAB1*) down-expressed in SS *vs* SC group, K7GPC8 (Leukocyte receptor LENG8), and P49932 (antibacterial peptide PMAP37) down-expressed in SR *vs* SS group, which may play the important regulatory roles in the process of piglet spleens in response to *C. perfringens* type C-infected diarrhea diseases.

GO enrichment analysis of DEPs suggested that the biological processes of immune response, regulation of cytokine, acute inflammatory response, programmed cell death, antimicrobial humoral response, apoptotic, defense response, transport and metabolic process were enriched. In addition, as shown in Fig. 5, the biological processes of the immune and inflammatory response might play core role in the development of piglet against *C. perfringens* type C infected diarrhea, as it could interact more with the remaining other biological processes. These results may hint that host immune responses were highly active against inflammation. Meanwhile, metabolic processes and transport were gradually complemented to supply the needs for materials and energy consumption, showing dynamic responses of piglet spleen during *C. perfringens* type C infection. Consistent with the biological process, the enrichment of cellular component and molecular function focused on immunoglobulin complex, oxidoreductase activity, hydrolase activity and immunoglobulin receptor binding, suggesting the transport and metabolism were active.

The protein abundance is the ultimate trait and functional phenotype. The down-regulated ALB (A0A287AMK0) and ORM1 (F1SN68) in SR *vs* SC group, the up-regulated HP (Q8SPS7) in SR *vs* SC, SR *vs* SS groups were the central hub of protein-protein interaction network, as they were both involved in protein biosynthesis, suggesting the potential functions of them participating in piglet resistant against diarrhea disease during *C. perfringens* type C infection. There were also many up-regulated key proteins were located in the node of network, such as LOC396684 (A0A287BM11), LOC100153899 (A0A287AVA9) and LOC106504547 (A0A287AJE3), through without characterized, they may also have some certain roles in the piglet resistant to *C. perfringens* type C infection, and it need to be explored in deep.

Haptoglobin (HP) is an acute phase reaction protein, it can play important roles in inhibiting bacteria, promoting angiogenesis, involving clearance of toxic and mediating host immune regulation (Griffiths et al., 2020). Study has been reported that HP not only can bind haemoglobin to counteract host inflammatory responses, but also interact with pro-inflammatory factor high mobility group box 1 (HMGB1) to elicit anti-inflammatory effects (VanPatten & Al-Abed, 2018; Wan et al. 2021). The differential expression of certain proteins may be the potential marker to discriminate pathological changes. Serum HP was significantly increased in chronic pancreatitis patients and pancreatic ductal adenocarcinoma patients, which was considered to be a potential biomarker for pancreatitis disease diagnosis (Ueda et al., 2016). Importantly, in this study, HP was all up-regulated in SR *vs* SC, SS *vs* SC and SS *vs* SR comparative groups, in the other word, *C. perfringens* infection caused the up-regulation of HP protein of piglets in SR and SS groups, meanwhile, the protein level of the susceptible piglets was higher than that in the tolerance piglets. The results may hint that HP was activated and involved in regulating the immune inflammatory response of piglet diarrhea, which may become a promising candidate marker for *C. perfringens* infection.

The most prominent differential expressed proteins among the DEPs identified in SR *vs* SC and SS *vs* SC were MXRA8, HP, S100A9, PMAP37, MX1, LENG8, and so on. Zhang et al. found that Matrix Remodeling Associated 8 (MXRA8) protein was a surface-exposed region and cellular receptor, anti-Mxra8 blocking antibodies may reduce the certain virus infection (Zhang et al. 2018), human monoclonal antibodies (mAbs) neutralize virus *in vitro* by preventing virus entry and spread and is protective *in vivo* in mouse models, which may have potential as a therapeutic agent or target of vaccine design against virus infections (Powell et al. 2020). The proteins S100A8, S100A9 and S100A12 were the members of calcium binding family S100, which pro-inflammatory alarmin associated with several inflammation-related diseases. They can bind to varieties of binding proteins or targets (such as enzymes, receptors, transcription factors, etc.) to regulate cell proliferation, induce cell apoptosis, participate in inflammatory response, and resist to pathogen invasion (Kerkhoff et al. 2005). Zhao et al. (2021) found that S100A9 protein was upregulated in the lung tissues of LPS-treated mice, inhibition of S100A9 protein alleviated LPS-induced lung injury. S100A9 protein blockade also attenuated the inflammatory responses and apoptosis in the lungs of LPS-challenged mice, S100A9 protein downregulation mitigated LPS-induced inflammation *in vitro* (Zhao et al. 2021), which were in accordance with our study, the S100A9 protein was expressed in piglet spleen tissue in SR and SS groups, the protein change level of S100A9 protein in SS group was higher than that in SR group, which may indicate that S100A9 protein could be considered as a potential candidate in infection disease.

Protein function can be predicted by using a database of protein domains and functional sites, in which, protein domain is applied to annotation of proteins with unknown function. Similarly, according to the enrichment of protein domain in Fig. 6, immunoglobulin subtype, heat shock protein and MHC class I-like antigen were significantly enriched in SR *vs* SC and SS *vs* SC, as well as GTPase domain and cytosolic fatty-acid binding. Further, many Immune inflammatory related functions were identified in SR *vs* SC, SS *vs* SC, and SS *vs* SR comparative groups, such as T-cell surface glycoprotein CD3, serotransferrin, serum albumin, MHC class I antigen 2 precursor, interferon-induced GTP-binding protein, lysozyme, haptoglobin.

Many studies have reported that PPAR-*γ* played a key role in mediating the anti-inflammatory effects (Mohamed et al. 2021; Choi et al., 2017; Chung et al. 2003). Specifically, the PPAR signaling pathway was the most significantly higher and activated in the SR and SS groups than SC group, the results suggested that PPAR signaling pathway was sustained to relatively high level, when resistant to *C. perfringens* infection. which may be a potential target against *C. perfringens* infection. Study had reported that PPAR- *γ* agonist seemed to enhance Fas-mediated apoptosis by affecting the way between caspase-8 and caspase-3, which was is also related to regulation of inflammation and cell proliferation (Chung et al. 2003). PPAR has the important regulation of cytokine production and cytokine-mediated signal transduction pathways in immune cells and cancer (Yang et al., 2008). Manoharan et al. (2016) had demonstrated that the PPAR pathway in innate immune cells orchestrates gut mucosal immunity and commensal homeostasis by regulating the expression of IL-22 and the antimicrobial peptides and calprotectin, suggesting that the PPAR signaling pathway played important innate immune function in regulating intestinal inflammation, mucosal immunity, and commensal homeostasis (Manoharan et al. 2016).

Further, according to bioinformatics comparison results, we identified many uncharacterized proteins (Table S3). These proteins identified possible genes by comparison. However, the bioinformatic analysis could not identify the roles of these proteins, and their functions remain unclear. Moreover, further studies of the molecular mechanism need to be performed to examine the underlying mechanism of the regulation of immune response networks and other biological processes.

## Conclusion

To summarize, we first analyzed the comparative proteomics in piglets after of *C. perfringens* infection, and found that PPAR signaling pathway and haptoglobin may play an important role in *C. perfringens* infection. This study offers information towards a deeper understanding of the immune inflammatory response of piglets to *C. perfringens* infection.

## Supplemental Information

10.7717/peerj.13006/supp-1 Supplemental Information 1 Workflow and strategy for quantitative proteomic analysis of *C. perfringens* infection for piglet diarrhea. Global proteome was performed in triplicate for the three groups in the spleen Note: SC, as control, represents the normal group without *C. perfringens* infection for piglets; SR means the resistant group after *C. perfringens* infection for piglets; while SS as the sensitive group after *C. perfringens* infection for piglets. The number from1 to 3 means three replicates in each group.

10.7717/peerj.13006/supp-2 Supplemental Information 2 Reproducibility of 3 repeated trials for SC, SR and SS. (A) PCA of the 3 biological repeats for the group of SC (red), SR (green) and SS (blue); (B) The boxplot plot of the relative standard deviation (RSD) of the quantitative values of proteins in each r

10.7717/peerj.13006/supp-3 Supplemental Information 3 Author checklist

10.7717/peerj.13006/supp-4 Supplemental Information 4 Supplemental Tables Table S1: The identified information of global proteome of SS, SR and SC groups by TMT. Table S2: The statistics of the i d en tified differential expressed proteins. Table S3: The annotation of uncharacterized proteins identified according to bioinformatics comparison results . Table S4: GO functional cat e g o ries of the DEPs among SS, SR and SC groups. Table S 5 : Protein functional domains of DEPs identified among SS, SR and SC groups . Table S6: KEGG signal pathway enrichment categories of the DEPs among SS, SR and SC groups. Table S7: PPI interaction network of the DEPs among SR, SS and SC groups.

## Additional Information and Declarations

### Data Availability

The following information was supplied regarding data availability: The data are available as Supplemental Tables.
