# Supplementary figures and images for: TMT labeled comparative proteomic analysis reveals spleen active immune responses during Clostridium perfringens type C infected piglet diarrhea
Source: PeerJ. 2022 Apr 4;10:e13006. doi: 10.7717/peerj.13006 (PMC8988937; doi:10.7717/peerj.13006)

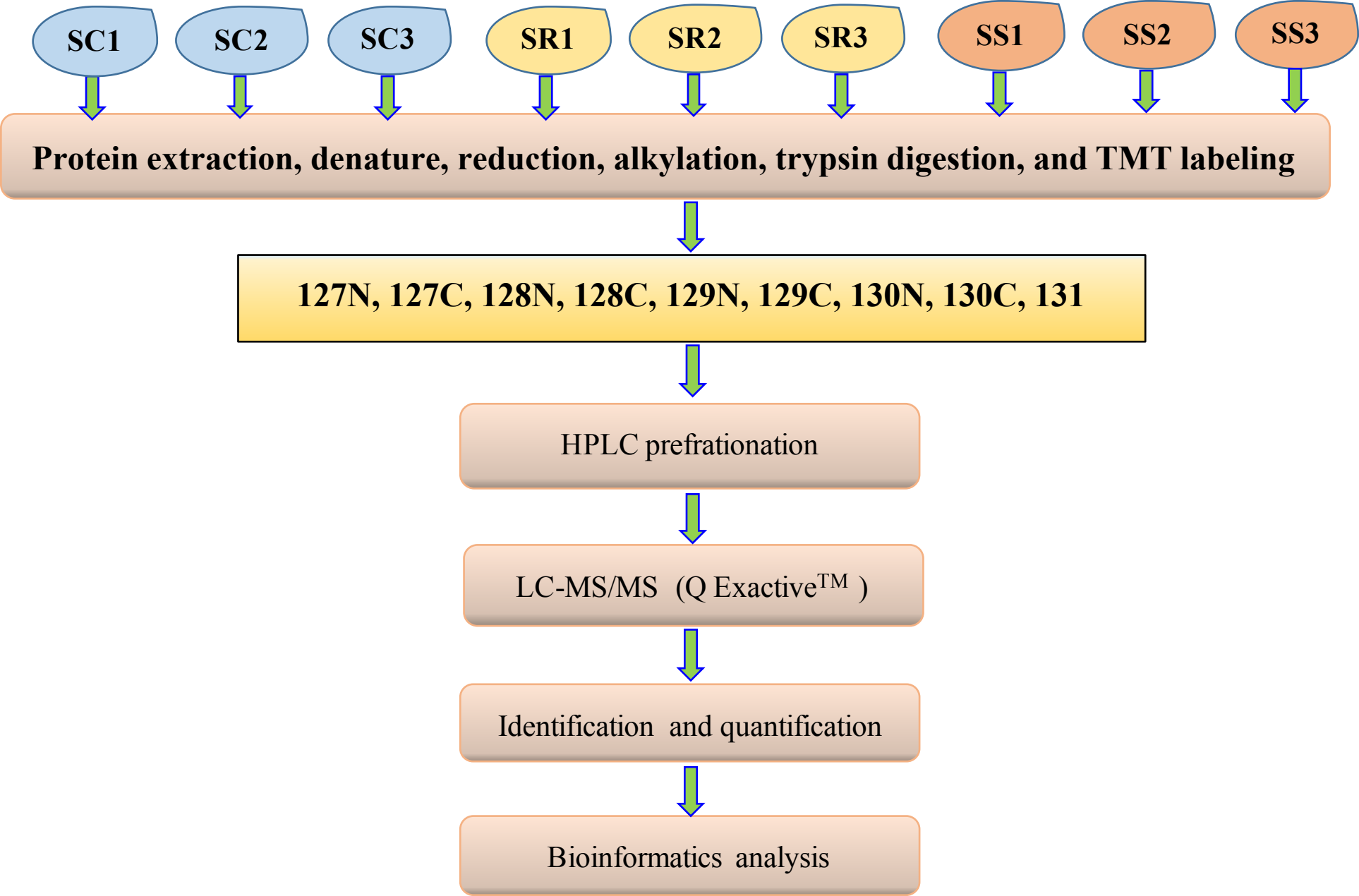

Supplement: Supplemental Information 1 — Note: SC, as control, represents the normal group without C. perfringens infection for piglets; SR means the resistant group after C. perfringens infection for piglets; while SS as the sensitive group after C. perfringens infection for piglets. The number from1 to 3 means three replicates in each group. [file peerj-10-13006-s001.pdf]

groups 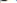 SC 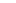 SR 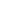 SS

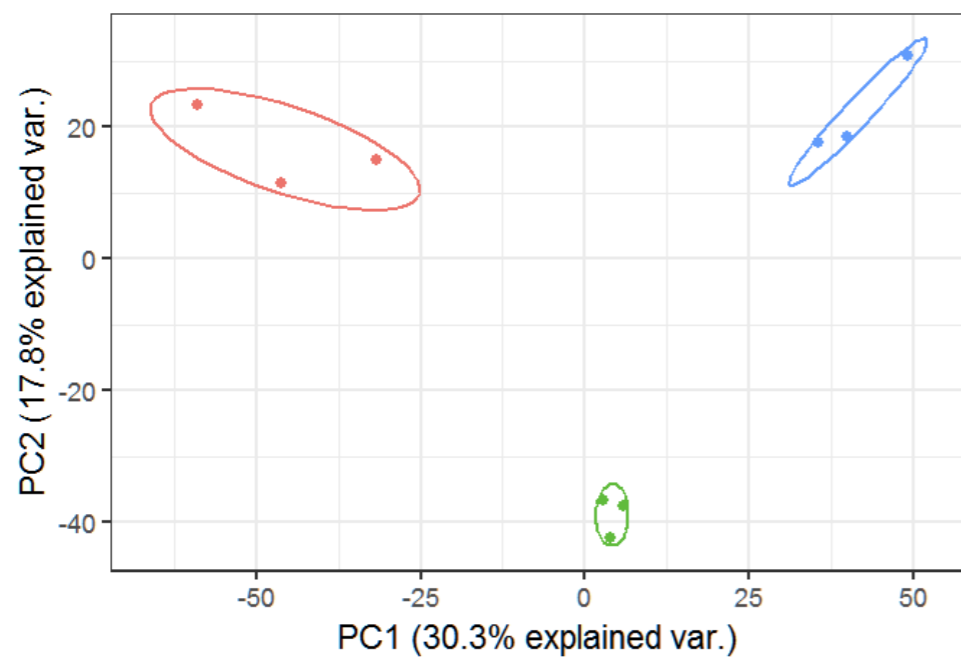

# B

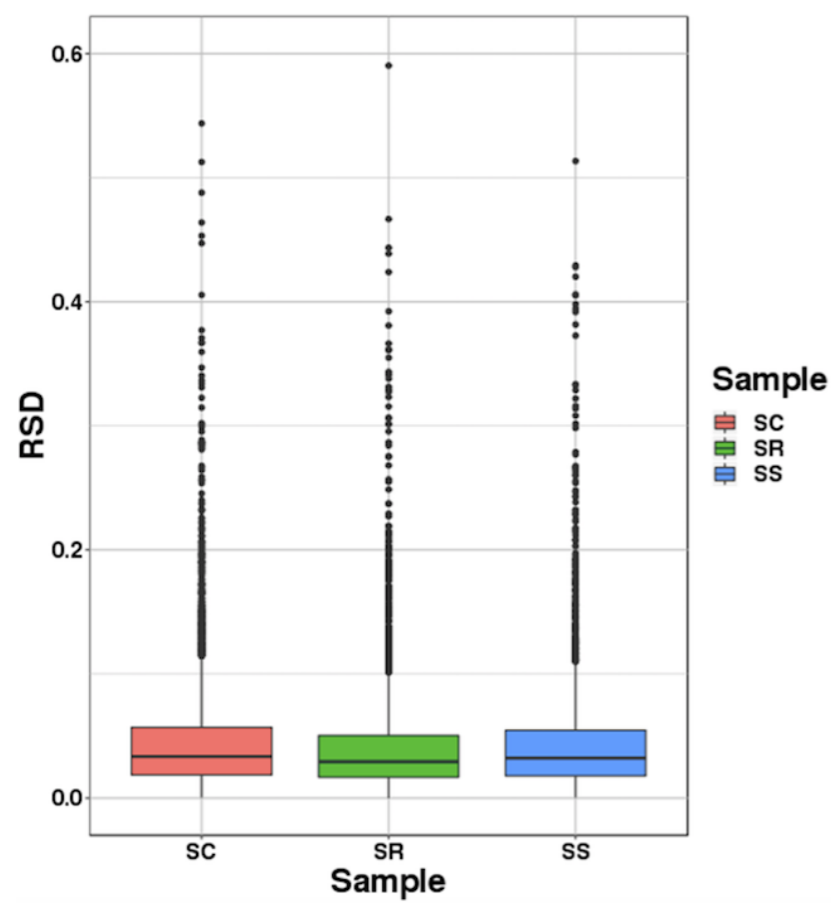

C

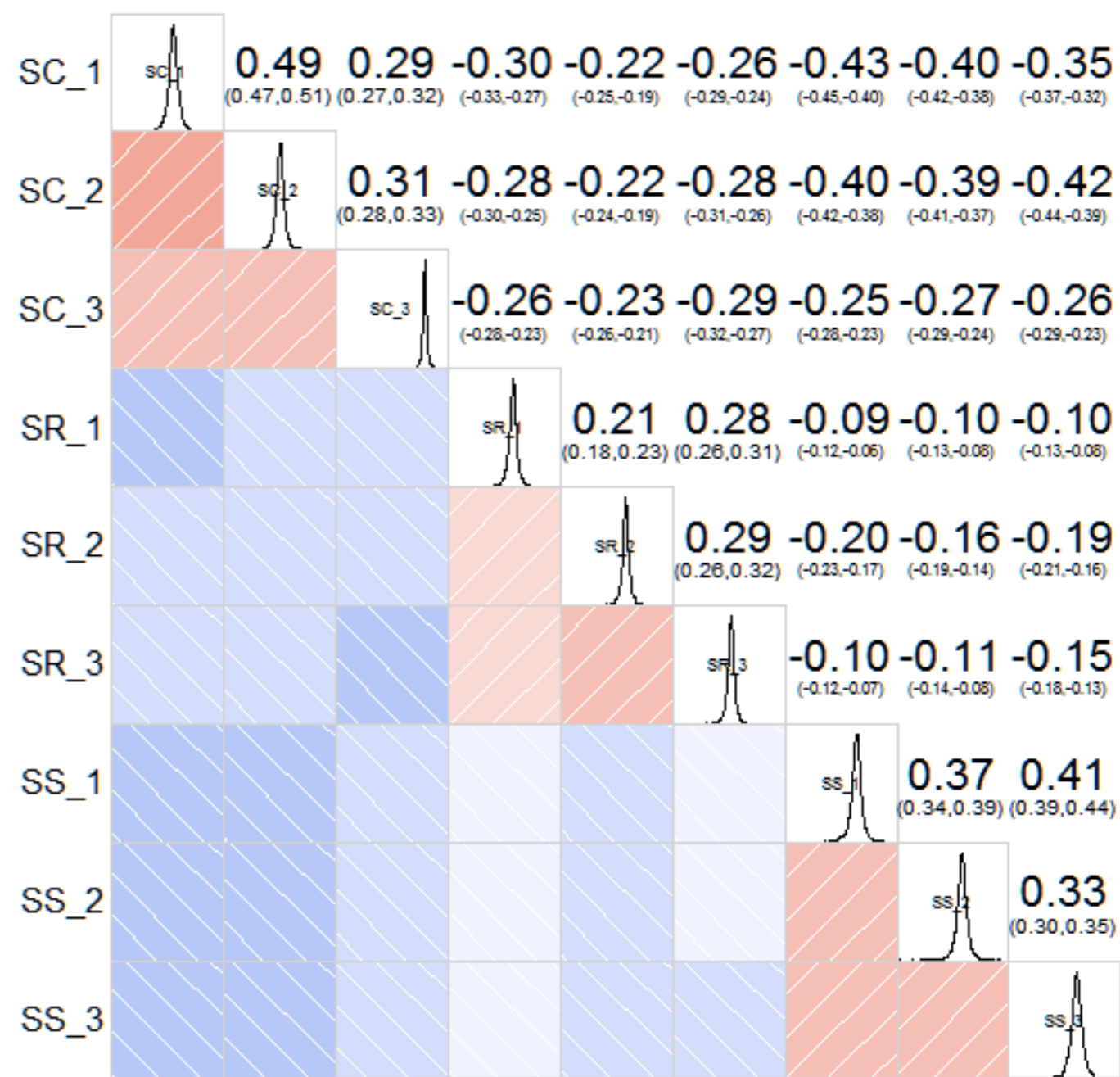

Supplement: Supplemental Information 2 [file peerj-10-13006-s002.pdf]
