# Supplementary material for: TMT labeled comparative proteomic analysis reveals spleen active immune responses during Clostridium perfringens type C infected piglet diarrhea
Source: PeerJ. 2022 Apr 4;10:e13006. doi: 10.7717/peerj.13006 (PMC8988937; doi:10.7717/peerj.13006)
Supplement: Supplemental Information 3 [file peerj-10-13006-s003.pdf]

NOTE: Please save this file locally before filling in the table, DO NOT work on the file within your internet browser as changes will not be saved. Adobe Acrobat Reader (available free here) is recommended for completion.

ARRIVE

The ARRIVE guidelines 2.0: author checklist

The ARRIVE Essential 10

These items are the basic minimum to include in a manuscript. Without this information, readers and reviewers cannot assess the reliability of the findings.

Item		Recommendation	Section/line number, or reason for not reporting
**Study design**	1	For each experiment, provide brief details of study design including:	Yes
		a. The groups being compared, including control groups. If no control group has been used, the rationale should be stated.	
		b. The experimental unit (e.g. a single animal, litter, or cage of animals).	three cages of animals
**Sample size**	2	a. Specify the exact number of experimental units allocated to each group, and the total number in each experiment. Also indicate the total number of animals used.	30 piglets for three cages
		b. Explain how the sample size was decided. Provide details of any *a priori* sample size calculation, if done.	according to the preliminary experiment
**Inclusion and exclusion criteria**	3	a. Describe any criteria used for including and excluding animals (or experimental units) during the experiment, and data points during the analysis. Specify if these criteria were established *a priori*. If no criteria were set, state this explicitly.	According to the obvious clinical symptoms of experimental piglets
		b. For each experimental group, report any animals, experimental units or data points not included in the analysis and explain why. If there were no exclusions, state so.	the piglets without obvious clinical symptoms were excluded
		c. For each analysis, report the exact value of *n* in each experimental group.	three groups of 5 piglets in each group
**Randomisation**	4	a. State whether randomisation was used to allocate experimental units to control and treatment groups. If done, provide the method used to generate the randomisation sequence.	Section Animal origin
		b. Describe the strategy used to minimise potential confounders such as the order of treatments and measurements, or animal/cage location. If confounders were not controlled, state this explicitly.	Section Piglets with C. perfringens infection
**Blinding**	5	Describe who was aware of the group allocation at the different stages of the experiment (during the allocation, the conduct of the experiment, the outcome assessment, and the data analysis).	Section Piglets with C. perfringens infection, Piglet sampling
**Outcome measures**	6	a. Clearly define all outcome measures assessed (e.g. cell death, molecular markers, or behavioural changes).	line 137-144
		b. For hypothesis-testing studies, specify the primary outcome measure, i.e. the outcome measure that was used to determine the sample size.	Not Applicable
**Statistical methods**	7	a. Provide details of the statistical methods used for each analysis, including software used.	Bioinformatic analysis of proteins<br>Statistical analysis
		b. Describe any methods used to assess whether the data met the assumptions of the statistical approach, and what was done if the assumptions were not met.	Statistical analysis
**Experimental animals**	8	a. Provide species-appropriate details of the animals used, including species, strain and substrain, sex, age or developmental stage, and, if relevant, weight.	Section Animal origin
		b. Provide further relevant information on the provenance of animals, health/immune status, genetic modification status, genotype, and any previous procedures.	Section Animal origin
**Experimental procedures**	9	For each experimental group, including controls, describe the procedures in enough detail to allow others to replicate them, including:	
		a. What was done, how it was done and what was used.	Materials & Methods
		b. When and how often.	Materials & Methods
		c. Where (including detail of any acclimatisation periods).	Materials & Methods
		d. Why (provide rationale for procedures).	
**Results**	10	For each experiment conducted, including independent replications, report:	Results
		a. Summary/descriptive statistics for each experimental group, with a measure of variability where applicable (e.g. mean and SD, or median and range).	
		b. If applicable, the effect size with a confidence interval.	line 280

The Recommended Set

These items complement the Essential 10 and add important context to the study. Reporting the items in both sets represents best practice.

Item		Recommendation	Section/line number, or reason for not reporting
Abstract	11	Provide an accurate summary of the research objectives, animal species, strain and sex, key methods, principal findings, and study conclusions.	line 23-41
Background	12	a. Include sufficient scientific background to understand the rationale and context for the study, and explain the experimental approach.	line 61-75,line
		b. Explain how the animal species and model used address the scientific objectives and, where appropriate, the relevance to human biology.	line 101-103
Objectives	13	Clearly describe the research question, research objectives and, where appropriate, specific hypotheses being tested.	line 61-75,line83-100
Ethical statement	14	Provide the name of the ethical review committee or equivalent that has approved the use of animals in this study, and any relevant licence or protocol numbers (if applicable). If ethical approval was not sought or granted, provide a justification.	line 105-109
Housing and husbandry	15	Provide details of housing and husbandry conditions, including any environmental enrichment.	line 134-138
Animal care and monitoring	16	a. Describe any interventions or steps taken in the experimental protocols to reduce pain, suffering and distress.	line 147
		b. Report any expected or unexpected adverse events.	no unexpected adverse events
		c. Describe the humane endpoints established for the study, the signs that were monitored and the frequency of monitoring. If the study did not have humane endpoints, state this.	line 147-149
Interpretation/ scientific implications	17	a. Interpret the results, taking into account the study objectives and hypotheses, current theory and other relevant studies in the literature.	Results
		b. Comment on the study limitations including potential sources of bias, limitations of the animal model, and imprecision associated with the results.	Discussion
Generalisability/ translation	18	Comment on whether, and how, the findings of this study are likely to generalise to other species or experimental conditions, including any relevance to human biology (where appropriate).	line 510-514
Protocol registration	19	Provide a statement indicating whether a protocol (including the research question, key design features, and analysis plan) was prepared before the study, and if and where this protocol was registered.	Acknowledgem ents
Data access	20	Provide a statement describing if and where study data are available.	Quantitative identification of spleen proteins for
Declaration of interests	21	a. Declare any potential conflicts of interest, including financial and non-financial. If none exist, this should be stated.	Declarations of interest
		b. List all funding sources (including grant identifier) and the role of the funder(s) in the design, analysis and reporting of the study.	Funding
